# Long-term impact of pulses and organic amendments inclusion in cropping system on soil physical and chemical properties

**DOI:** 10.1038/s41598-023-33255-3

**Published:** 2023-04-20

**Authors:** C. P. Nath, Asik Dutta, K. K. Hazra, C. S. Praharaj, Narendra Kumar, S. S. Singh, Ummed Singh, Krishnashis Das

**Affiliations:** 1 Division of Crop Production, ICAR-Indian Institute of Pulses Research, Kanpur, 208 024 India; 2 ICAR-Directorate of Groundnut Research, Junagadh, Gujarat 362 001 India; 3 Rani Lakshmi Bai Central Agricultural University, Jhansi, 284 003 India; 4 Agriculture University, Jodhpur, Rajasthan 324 001 India; 5 ICAR-Indian Agricultural Research Institute, Pusa, New Delhi 110 012 India

**Keywords:** Agroecology, Ecology

## Abstract

Mono-cropping of maize–wheat, mechanical disintegration of soils, and continuous chemical fertilization have deteriorated soil health in the Indo-Gangetic Plains. We studied the long-term impact of pulse-based cropping systems with integrated nutrient management on soil physical and chemical properties and yield sustainability. We evaluated four different cropping systems: (1) maize–wheat (M–W), (2) maize–wheat–mungbean (M–W–Mb), (3) maize–wheat–maize–chickpea (M–W–M–C), (4) pigeonpea–wheat (P–W) each with three degrees of soil fertilization techniques: (1) unfertilized control (CT), (2) inorganic fertilization (RDF), and (3) integrated nutrient management (INM). The field experiment was undertaken in a split-plot design with three replications each year with a fixed layout. P–W and M–W–Mb systems enhanced soil properties such as volume expansion by 9–25% and porosity by 7–9% (*p* < 0.05) more than M–W, respectively. P–W and M–W–Mb increased soil organic carbon by 25–42% and 12–50% over M–W (RDF). P–W system enhanced water holding capacity and gravimetric moisture content by 10 and 11% (*p* < 0.05) than M–W. Pulse-based systems (P–W and M–W–Mb) had higher available nitrogen (8–11%), phosphorus (42–73%), and potassium (8–12%) over M–W (*p* < 0.05). M–W–Mb increased 26% maize yield and 21% wheat yield over M–W (*p* < 0.05) at the thirteenth crop cycle. P–W system had a higher sustainable yield index (*p* < 0.05) of wheat over the M–W. Thus, pulse inclusion in the cropping system in combination with INM can enhance physical and chemical properties vis-à-vis sustainable yield index over the cereal-cereal system.

## Introduction

Soil physical properties are a significant part of the soil system working and empower to assess the ecosystem sustainability^1, 2^. The physical properties of soil as bulk density, soil structure, and water-holding capacity are signs of good soil well-being in the long-run^3^. The water-holding capacity of soil is regulated by: (1) pore size distribution, (2) surface area of soil, and (3) aggregate stability. Fundamentally, soil aggregates (macro–and micro–aggregates) minimize organic matter mineralization, sequestering soil organic carbon (SOC) and nutrients^4^. Theoretically, increased water-holding capacity, soil moisture content, and aggregate stability are indicators of the positive impact of crop diversification and balanced fertilization on soil health^1, 5^. Soil aggregated nutrients strongly influenced by crop management practices that include tillage/mechanical disruption, crop rotation, and fertilization techniques^6^. Rice–wheat (9.64 m ha area) and maize–wheat (1.83 m ha area) are the two dominant cropping systems in the Indo-Gangetic plains (IGP)^6, 7^. These cropping systems reduced crop productivity and soil ecology because of SOC depletion, macro- and micro-nutrient deficiency, the decline in soil microbial/biological properties, and groundwater table depletion^8, 9^. Pulse crops in the cropping system might reduce soil health deterioration and increase yield sustainability^9^. A long-term study indicated that the chickpea yield was more sustainable than the maize^10^. Therefore, it is necessary to evaluate the long-term impact of pulses in cropping systems on soil physical and chemical properties and crop yields in the IGP.

Soil water holding capacity has a significant role in the soil–plant–atmospheric water balance. Water holding capacity is regulated by various soil physico-chemical and biological indices^11^. However, the effects of soil factors on water-holding capacity cannot be generalized across regions. For example, Khaleel et al. saw that 80% of fluctuation in water-holding capacity was because of texture and carbon^12^. While a meta-analysis study reported a mean increase of only 1.2% volumetric available water capacity with a 1% mass increase in soil organic carbon^11^. However, these studies stated a definite impact of soil carbon on water-holding capacity. Hence, the management-induced changes in carbon content eventually change the water-holding capacity and crop productivity over time. Evaluation of crop rotation with diverse crop phenology as rooting behavior of cereals, pulses, and biomass accumulation is of great value to designing a sustainable cropping intensification^13^. Previous studies reported the beneficial effects of pulse crops on biological nitrogen fixation and carbon dynamics^4, 14, 15^. The added residues of pulse crops provide macro- and micro-nutrients to the soil that enhance soil fertility, improve soil aggregates, and encourage adaptive capacity of plants to adverse environments^16^. For instance, an eight-year study from Alberta in Canada stated that pulse-based rotation (pea-wheat) increased SOC from a baseline level of 10.3–11.2 g kg^−1^ which was higher than wheat monoculture^17^. Also, a pulse-based intensive cropping system increases biomass return into the soil and carbon sequestration^18^. Despite increments in SOC under pulse-based cropping systems, but long-term impacts of pulses in cropping systems on soil physical and chemical functions are less reported in the subtropical climate.

Nutrient management practices influence soil health by altering physical and chemical properties of soil in the long-run. Hence, inappropriate nutrient management results in decline in crop productivity and deterioration in soil health^19^. Organic amendments retain soil moisture, increasing soil infiltration and favoring crop growth and yield^20^. Besides, the addition of organic amendments (farmyard manure + crop residues) in soil not only proliferates the microbial density in rhizospheric soil but also enhances the microbial diversity in rhizospheric soil. Thus, it can support soil structure-building processes through favorable interaction between soil aggregate and organic matter^21^. Results from Sanborn University of Missouri (Columbia) after 100 years of corn-wheat-red clover rotation depicted that manure addition decreased bulk density (by 0.12 g cm^−3^) and increased saturated hydraulic conductivity (by 9 times) than unfertilized control^22^. A long-term study of 5 years from vertisol of India indicated that integrated nutrient management (INM) comprising chemical fertilizers and farm-yard manure (5 Mg ha^−1^) increased 20.9% and 13.1% mean grain yield of maize and chickpea, respectively over chemical fertilization^10^. An 18 years study with a rice–wheat cropping system in a *Typic Haplustept* of Indian IGP deciphered that INM (chemical fertilizers + farm-yard manure + crop residues) enhanced soil aggregation, aggregate-associated carbon, and carbon stock^23^. Mean weight diameter, commonly used to express aggregate stability, was higher in manure application than in control treatments^24^. An improved understanding of soil physical and chemical properties and yields of crops under different cereal-pulse rotations with fertilization techniques will help to intensify cropping systems with pulse crops in a sustainable manner^10^. However, the impact of pulses and organic amendments in systems on bulk density and associated soil physical properties and crop productivity is less studied in the subtropical IGP^1^.

In this regard, long–term experiments provide valuable information on management-induced alteration of physical and chemical properties of soil^25^. Maize–wheat–red clover significantly increased soil strength, aggregate stability, and yields than wheat–maize rotation in Missouri (Columbia)^22^. Effects of cropping system/fertilizer management on soil physical properties are affected by soil type, antecedent soil properties, climate, land use, and soil structure formation process^20^. Pulse crops provide annual input of biological N fixation at ~ 3 Tg enhancing the SOC concentration and overall soil health^26^. It is necessary to assess the regional-specific soil properties under variable crop management for yield sustainability. The degraded cultivated soils must be urgently restored in IGP using sustainable practices and adapted soil management strategies. Therefore, an integrated assessment of soil quality and crop yield is needed for crop management practices for agricultural sustainability in the region.

Therefore, the present study focused on two objectives: (1) to evaluate the long-term impact of pulses in the cropping system and fertilization techniques on soil physical and chemical properties and yields of component crop/system, (2) to determine the effects of pulse-based cropping systems with INM on soil quality as indicated by water holding capacity, moisture content, bulk density, water-filled pore space, aggregated properties (aggregates fraction and aggregated N and P content), SOC and their relations with crop yield. We hypothesized that soil properties and crop productivity improved in pulse-based cropping systems with INM compared with maize–wheat monoculture and chemical fertilization.

## Materials and methods

### Site characteristics

The present long-term experiment started in 2003 at the ICAR-Indian Institute of Pulses Research, Kanpur (26° 27′ N latitude and 80° 14′ E longitude), India. The trial region belongs to subtropical climate having a mean yearly air temperature of 26.0 °C. Mean annual rainfall was around 722 mm during 2004–2017. The least precipitation (510 mm) was in 2015, and the maximum (1225 mm) was in 2013. The soil order of the study site is *Inceptisol* (Typic Ustochrept). Soil properties (sandy-loam texture) at the start of the experiment at 0–15 cm depth included: pH 8.1 (soil-to-water proportion of 1:2.5), 2.8 g kg^−1^ SOC, 182 kg ha^−1^ available nitrogen (N), 18.6 kg ha^−1^ phosphorus (P) and 159 kg ha^−1^ potassium (K).

### Treatment details and layout

The experimental design was split-plot with three replications every year. The experiment consisted of four cropping systems (main plot): maize–wheat (M–W), maize–wheat–mungbean (M–W–Mb), maize–wheat–maize–chickpea (M–W–M–C) and pigeonpea–wheat (P–W) and three soil fertilization techniques (subplot): no-fertilizer application (CT), recommended chemical fertilizers of the region (RDF), and integrated nutrient management [INM: 50% fertilizer dose of RDF + full crop residues + farmyard manure 5 t ha^−1^ + biofertilizers]^14^. Each subplot size was 49 m^2^ (7 m × 7 m). Thus, we studied total number of 36 plots (3 replications × 4 different crop rotations × 3 fertilization treatments = 36). Farmyard manure was mixed uniformly during land preparation (with tillage) 2 weeks before sowing during the rainy season. The farmyard manure contained 0.56% nitrogen, 0.18% phosphorus, and 0.52% potassium. Biofertilizers (*Azotobacter* for maize and wheat, *Rhizobium* for pigeonpea, chickpea, mungbean, and phosphate solubilizing bacteria *Bacillus polymyxa* for all crops) were applied (10^7^ bacteria g^−1^ culture) through seed treatment at the time of sowing. Fertilizer doses were 120:60:40 kg ha^−1^ N:P_2_O_5_:K_2_O (maize and wheat) and 20:60:40 kg ha^−1^ of N:P_2_O_5_:K_2_O (for pulses) in RDF. Fertilizers were applied in three splits with the one-third amount of N (through urea), the full rate of P (through diammonium phosphate), and K (through muriate of potash) at sowing and remaining at 25 and 45 days after sowing (DAS) in cereals. All fertilizers were applied during sowing in pulse crops.

### Crop management

The cultivars were ‘Azad Uttam’ for maize, ‘UPAS 120’ for pigeonpea, ‘PBW 343’ for wheat, ‘DCP 92-3’ for chickpea, and ‘Samrat’ for mungbean. The seasons consisted of June to October as the rainy (maize and pigeonpea), November to March as the winter (wheat and chickpea), and April to June as the summer (mungbean). The seed rates included 20 kg ha^−1^ for maize, 15 kg ha^−1^ for pigeonpea, 100 kg ha^−1^ for wheat, 80 kg ha^−1^ for chickpea, and 12 kg ha^−1^ for mungbean. The required irrigations were two for maize/chickpea/pigeonpea, five for wheat, and four for mungbean. On average (average of 14 years), the applied amount of irrigation water was 525 mm in maize, 380 mm in wheat, 225 mm in pigeonpea and chickpea, and 256 mm in mungbean, irrespective of treatments.

The yield data of all component crops in each system was presented for four cropping cycles (2013–2014 to 2016–2017). The yields of 4 years represent the 11th–14th cycles of experimentation. Wheat was the common crop in each rotation in the present study. Hence, we used base–crop (wheat) productivity and sustainable yield index as indicative for the assessment of soil health^27^. A net plot area of 5 m × 5 m was manually harvested for seed yield estimation in each crop and expressed as t ha^−1^ at 14% moisture.

### Soil sampling, processing and analysis

The soil was collected after 14 years (April 2017) at the harvest of the wheat crop because wheat was the base crop for all systems. Soil samples were collected at the same time for the present study. The requirement of soil sampling and processing differed for various parameters under study. Accordingly, we elaborated the sampling procedure subsequently. For example, the soil was collected from six sites in each plot (~ treatment) from each replication at two depths (0–20 cm and 20–40 cm). Soil sampling was performed with a post-hole auger (having a sharp edge at the end to open the pit at the sampling site) with a core height of 20 cm for analysis of physical indices. A composite sample was prepared by mixing the collected soil from each plot^4^. We analyzed 36 samples for each parameter based on the design of the experiment (4 cropping systems × 3 fertilization techniques × 3 replications) for affirming the exactness of the results. The composite soil was separated into two sub-sets. One sub-set was air-dried for 72 h and passed through a 2.0 mm sieve and oven-dried at 105 °C for 24 h for examination of soil physical and chemical properties. Another sub-set (field-moist soil) was sieved with a 3 mm screen and kept in packed plastic bags at 4 °C for soil biological properties assessment. It was analyzed within seven days of sampling.

### Bulk density, specific volume and total porosity

Soil sampling was performed with three undisturbed soil cores at two depths (0–20 cm and 20–40 cm). A core sampler with a core height of 12.6 cm and a 2.45 cm radius was used for dry bulk density estimation with the method described by Veihmeyer and Hendrickson^28^. The core was inserted into the soil with a hammer for sampling without disturbing the soil block. The sampled soil blocks were trimmed to the precise rim/volume of the core and oven-dried at 105 °C for 24 h^28^. A particle density value of 2.65 g cm^−3^ was considered for porosity calculation^28^. Dry bulk density was calculated using Eq. (1) below:

1 $${\text{Dry}}\;{\text{bulk}}\;{\text{density}}\left( {{\text{g}}\;{\text{cm}}^{{ - {3}}} } \right) = \frac{{{\text{M}}_{{\text{d}}} }}{{\text{V}}}$$

where, M_d_ is the weight of dry soil (g), V is the volume of soil (cm^3^)

Specific volume (Eq. 2) of soil was calculated by formula of Veihmeyer and Hendrickson^28^:

2 $${\text{Specific volume }}({\text{cm}}^{3} {\text{g}}^{{ - 1}} ) = \frac{{{\text{Total volume of soil }}({\text{cm}}^{3} )}}{{{\text{Mass of dry soil sample (g)}}}}$$

Subsequently, total porosity was calculated using Eq. (3) below:

3 $${\text{Total porosity }}(\% ) = \left( {{\text{1}} - \frac{{{\text{bulk density}}}}{{{\text{particle density}}}}} \right) \times 100$$

Subsequently, different ratios were calculated by using formulas given by Das and Agrawal ^29^ as given below:

4 $${\text{Void ratio }} = \frac{{{\text{Porosity }}(\% )}}{{{\text{100}} - {\text{porosity }}(\% )}}$$

5 $${\text{Air}}\;{\text{ratio}} = \frac{{{\text{Volume}}\;{\text{of}}\;{\text{air}}}}{{{\text{Volume}}\;{\text{of}}\;{\text{solid}}}}$$

6 $${\text{Liquid}}\;{\text{ratio}} = \frac{{{\text{Volume}}\;{\text{of}}\;{\text{water}}}}{{{\text{Volume}}\;{\text{of}}\;{\text{solid}}}}$$

7 $${\text{Volume}}\;{\text{of}}\;{\text{solid}} = {\text{Total}}\;{\text{volume}}{-}\left( {{\text{volume}}\;{\text{of}}\;{\text{water}} + {\text{volume}}\;{\text{of}}\;{\text{air}}} \right)$$

### Water holding capacity and volume expansion

Soil water holding capacity was estimated using Keen Raczkowski box by placing soil samples on a porous plate with applying pressure to drain water to field capacity using the method of Saha^30^. Keen Raczkowski Box (5.6 cm internal diameter and 1.6 cm height) had a perforated base with holes of 0.75 mm diameter and spaced 4 mm apart. The perforated base of the cylinder was covered with filter paper and filled with soil up to the top edge of the Keen Raczkowski box. The box was weighed and placed in a tray. Water was added to the tray and left overnight for equilibrium. At equilibrium, the soil was fully saturated and the box was removed from the tray. The expanded wet soil found above the rim of the box was removed using the straight edge of a spatula. The removed soil was kept in a pre-weighed aluminium moisture box. The Keen Raczkowski box and aluminium moisture box were weighed immediately and then oven-dried at 105 °C for 24 h to dry the soil^14^. The dry weight of both boxes was recorded. The matric potential at the water holding capacity measurement varied between (−) 90.8 and (−) 114.1 kPa at 0–20 cm and (−) 109.3 to (−) 116.4 kPa at 20–40 cm.

The water holding capacity and volume expansion were calculated using equations given by Reynolds^31^:

8 $${\text{Water holding capacity }}(\% ) = \frac{{{\text{Weight of wet saturated soil }}({\text{g}}) - {\text{Weight of total oven dry soil }}({\text{g}})}}{{{\text{Weight of total oven dry soil }}({\text{g}})}} \times 100$$

9 $${\text{Volume expansion }}({\text{cm}}^{3} ) = \frac{{{\text{Volume of expanded soil}}}}{{{\text{Volume of air dry soil}}}}$$

### Gravimetric and volumetric moisture content

Gravimetric moisture content was determined by method of Reynolds^31^ by collecting field moist soils with soil cores at two depths (0–20 cm and 20–40 cm). The collected soil sample was immediately kept in an aluminium moisture box (weighed moisture box) and wrapped with a cotton cloth to protect it from evaporation. The moisture boxes were transferred to the laboratory after sampling and fresh weight was recorded. Subsequently, an aluminium box filled with soil was oven-dried at 105 °C for 72 h. The weight of aluminum boxes was deducted from the fresh and dry weight of the sample in the respective treatment. Finally, gravimetric moisture content and volumetric moisture content were estimated using formulae of ^31^:

10 $${\text{Gravimetric moisture content }}(\% ) = \frac{{{\text{Wet weight of soil sample }}({\text{g}}) - {\text{Dry weight of soil sample }}({\text{g}})}}{{{\text{Dry weight of soil sample }}({\text{g}})}} \times 100$$

11 $$\begin{aligned} & {\text{Volumetric}}\;{\text{moisture}}\;{\text{content}}\;\left( \% \right) \\ & \quad = {\text{Gravimetric}}\;{\text{moisture}}\;{\text{content}} \times {\text{Corresponding}}\;{\text{dry}}\;{\text{bulk}}\;{\text{density}}\;{\text{of}}\;{\text{soil}}\;{\text{for}}\;{\text{each}}\;{\text{treatment}}. \\ \end{aligned}$$

The air-filled porosity and water-filled pore space were calculated using formulas of Das and Agrawal^29^:

12 $${\text{Air-filled}}\;{\text{porosity}} = {\text{Total}}\;{\text{porosity}} - {\text{Volumetric}}\;{\text{moisture}}\;{\text{content}}$$

13 $${\text{Water-filled pore space }}(\% ) = \frac{{{\text{Volumetric moisture content}}}}{{{\text{Total porosity}}}} \times 100$$

### Aggregate stability and aggregate associated N/P estimation

The wet sieving technique was used for soil aggregation following the standards of Yoder's apparatus through a progression of four sieves (2, 0.5, 0.25, and 0.053 mm)^1, 4, 8, 32, 33^. A 100 g of > 4 mm soil aggregates were put on top of a 2 mm sieve. Yoder's apparatus comprised a water drum that was loaded with deionized water. The sieving process finished by moving the sieves all over 3 cm in deionized water with a frequency of 25 times each minute in this water drum. The soil was moved upward in a water drum for 5 min. The sieving system brought about the development of four total size portions: (1) > 2 mm (coarse macroaggregates), (2) 0.25–2 mm (macroaggregates), (3) 0.053–0.25 mm (coarse macroaggregates), and (4) < 0.053 mm (‘silt + clay’ − size particles). Soil material held on each sieve after wet sieving was moved into a container and dried at 65 °C until a steady weight^32^.

14 $$\begin{aligned} {\text{Water}}\;{\text{stable}}\;{\text{macroaggregates}}\;\left( { > 0.{25}\;{\text{mm}}} \right) & = {\text{coarse}}\;{\text{macroaggregates}}\;\left( { > {2}\;{\text{mm}}} \right) \\ & \quad + {\text{mesoaggregates}}\;\left( {0.{25}{-}{2}\;{\text{mm}}} \right). \\ \end{aligned}$$

15 $$\begin{aligned} {\text{Water}}\;{\text{stable}}\;{\text{microaggregates}}\;\left( { < 0.{25}\;{\text{mm}}} \right) & = {\text{coarse}}\;{\text{microaggregates}}\;\left( {0.0{53}{-}0.{25}\;{\text{mm}}} \right) \\ & \quad + {\text{silt}} + {\text{clay}}\;{\text{fractions}}\;\left( { < 0.0{53}\;{\text{mm}}} \right). \\ \end{aligned}$$

Mean weight diameter was calculated with Van Bavel and Kirkham method^34^:

16 $${\text{Mean}}\;{\text{weight}}\;{\text{diameter}}\; = \sum\limits_{{{\text{i}} = {1}}}^{{\text{n}}} {{\text{xi}} \cdot {\text{wi}}}$$

xi is the mean diameter of the i-th size class (mm), and wi is the proportion of the total sample in the corresponding size fraction.

The available N and P content in water-stable macroaggregates and water-stable microaggregates were estimated using the alkaline permanganate procedure for N^35^ and Olsen method for P^36^ and expressed in mg kg^−1^ dry soil.

### Soil organic carbon, available nutrients, and biological properties (bulk soil)

The estimation methods were wet oxidation strategy for soil organic carbon^37^, Alkaline KMnO_4_ technique for available N^35^, Olsen's extractant for available P (0.5 N NaHCO_3_, pH 8.5)^36^, and 1 N NH_4_OAc for available K (pH 7.0)^38^. Soil pH (soil-to-water proportion of 1:2.5) was assessed by techniques of Jackson ^38^. The chloroform-fumigation extraction technique was used for microbial biomass carbon and communicated as mg kg^−1^ dry soil^39^. The extraction efficiency of microbial biomass carbon (kEC) was 0.45^39^. Alkaline phosphatase was determined using 16 mM para (p)-nitrophenyl phosphate as substrate and reported as µg p-nitrophenol produced g^−1^ soil h^−1^ ^40^. The β-glucosidase was assessed utilizing 25 mM p-nitrophenol-β-d-glucopyranoside as the substrate^41^.

### Yield estimation

Yields of each crop were converted to wheat equivalent yield using price of crops^6^. The sum of wheat yields and wheat equivalent yields of other crops was the system productivity as follows:

17 $${\text{Wheat}}\;{\text{equivalent}}\;{\text{yield}}\;{\text{of}}\;{\text{maize}} = \left[ {\left( {{\text{Maize}}\;{\text{yield}} \times {\text{price}}\;{\text{of}}\;{\text{maize}}} \right)/\left( {{\text{price}}\;{\text{of}}\;{\text{wheat}}} \right)} \right]$$

18 $${\text{Wheat}}\;{\text{equivalent}}\;{\text{yield}}\;{\text{of}}\;{\text{pigeonpea}} = \left[ {\left( {{\text{Pigeonpea}}\;{\text{yield}} \times {\text{price}}\;{\text{of}}\;{\text{pigeonpea}}} \right)/\left( {{\text{price}}\;{\text{of}}\;{\text{wheat}}} \right)} \right]$$

19 $${\text{Wheat}}\;{\text{equivalent}}\;{\text{yield}}\;{\text{of}}\;{\text{chickpea}} = \left[ {\left( {{\text{Chickpea}}\;{\text{yield}} \times {\text{price}}\;{\text{of}}\;{\text{chickpea}}} \right)/\left( {{\text{price}}\;{\text{of}}\;{\text{wheat}}} \right)} \right]$$

20 $${\text{Wheat}}\;{\text{equivalent}}\;{\text{yield}}\;{\text{of}}\;{\text{mungbean}} = \left[ {\left( {{\text{Mungbean}}\;{\text{yield}} \times {\text{price}}\;{\text{of}}\;{\text{mungbean}}} \right)/\left( {{\text{price}}\;{\text{of}}\;{\text{wheat}}} \right)} \right]$$

Sustainable yield index of wheat was calculated as follows^42^.

21 $$\text{SYI }= \frac{Y-\sigma }{{Y}_{max}}$$

where, *Y* is the estimated average yield of base-crop across the years; *σ* is its estimated standard deviation, and *Y*_*max*_ is the observed maximum yield of base-crop.

### Statistical analysis

The analysis of data was performed by the analysis of variance (ANOVA) technique for split-plot design utilizing the program OPSTAT^43^. For mean comparison, Tukey's honest significance test was used at *p* ≤ 0.05. The bivariate regression among wheat yield and chosen soil parameters were undertaken by Microsoft Excel™ 2007^44^.

## Results

### Bulk density, void ratio, and air-filled porosity

P–W system decreased bulk density (by 0.06 g cm^−3^) compared to the M–W system at 0–20 cm (*p* < 0.05) (Table 1). Notably, M–W–M–C and P–W systems had lower bulk density (mean 4%) than the cereal-cereal system (M–W) (*p* < 0.05). P–W and M–W–Mb systems significantly increased void ratio and air-filled porosity over M–W (Table 1; Supplementary Table S1). The P–W rotation enhanced 5–19% void ratio and 25–54% air-filled porosity over M–W across depth (*p* < 0.05). Long-term practice of INM had reduced dry bulk density more than RDF by 3% (*p* < 0.05).

**Table 1:** Impact of pulses and organic amendments on soil physical indices in long-run.

Depth	Cropping system	Bulk density (g cm^−3^)	Void ratio	Water-filled pore space (%)	Air ratio
0–20 cm	M–W	1.28 ± 0.01^a#^	0.97 ± 0.003^b^	91.6 ± 0.45^a^	0.15 ± 0.011^b^
	M–W–Mb	1.26 ± 0.03^a^	1.09 ± 0.023^a^	90.5 ± 0.40^a^	0.19 ± 0.002^a^
	M–W–M–C	1.23 ± 0.04^b^	1.14 ± 0.019^a^	83.7 ± 1.63^b^	0.19 ± 0.008^a^
	P–W	1.22 ± 0.02^b^	1.15 ± 0.012^a^	87.1 ± 0.86^ab^	0.20 ± 0.003^a^
	*Nutrient management*				
	CT	1.28 ± 0.02^a^	1.04 ± 0.001^b^	85.1 ± 0.31^b^	0.15 ± 0.003^c^
	RDF	1.25 ± 0.01^ab^	1.08 ± 0.012^b^	89.4 ± 0.22^a^	0.17 ± 0.007^b^
	INM	1.21 ± 0.01^b^	1.14 ± 0.008^a^	90.3 ± 1.08^a^	0.23 ± 0.001^a^
	Interaction	*	*	NS	*
20–40 cm	*Cropping system*				
	M–W	1.36 ± 0.04^a^	1.11 ± 0.012^b^	92.3 ± 0.21^a^	0.09 ± 0.001^b^
	M–W–Mb	1.32 ± 0.01^b^	1.17 ± 0.011^a^	89.8 ± 0.30^bc^	0.11 ± 0.003^a^
	M–W–M–C	1.31 ± 0.01^b^	1.18 ± 0.004^a^	90.9 ± 0.51^ab^	0.11 ± 0.004^a^
	P–W	1.33 ± 0.03^b^	1.17 ± 0.003^a^	89.2 ± 0.32^c^	0.12 ± 0.002^a^
	*Nutrient management*				
	CT	1.36 ± 0.05^a^	1.10 ± 0.010^c^	92.3 ± 0.11^a^	0.09 ± 0.001^b^
	RDF	1.33 ± 0.04^b^	1.16 ± 0.004^b^	91.2 ± 0.22^b^	0.10 ± 0.003^b^
	INM	1.30 ± 0.02^c^	1.21 ± 0.003^a^	88.2 ± 0.16^c^	0.14 ± 0.001^a^
	Interaction	*	*	*	*

^#^Lowercase letters (a–c) after values (mean ± standard error) delineates significant difference at *p* ≤ 0.05 using Tukey's honest significance test; *denotes interaction is significant; NS = non-significant.

### Water filled pore space, liquid ratio and air ratio

M–W–M–C and P–W had a lower water-filled pore space (by 5–9%) and liquid ratio (4–10%) than M–W across depth (Table 1; Supplementary Table S1). Subsequently, pulse-based systems had a significantly higher air ratio (0.2) over M–W (0.15). Even in lower soil depth (20–40 cm), P–W and M–W–Mb systems decreased water-filled pore space and increased air ratio compared with M–W (*p* < 0.05). INM minimized 5% liquid ratio and increased 33–53% air ratio (*p* < 0.05) compared with RDF across depths (Table 1; Supplementary Table S1).

### Soil mass-volume relationship

P–W system had the higher water holding capacity and moisture content (gravimetric and volumetric) by 9, 10, and 5% (p < 0.05) higher than the M–W system, respectively in 0–20 cm depth (Table 2). The extent of increase was 6, 7, and 4% under the P–W system at 20–40 cm depth over the M–W. INM increased 6–7% gravimetric moisture content, 6–7% water holding capacity, and 4–5% volumetric moisture content over RDF (Table 2). P–W (INM) and M–W–Mb (INM) increased 6–18% water holding capacity (*p* < 0.05) and 5–21% gravimetric moisture content over M–W (RDF) at 20–40 cm depth (Supplementary Fig. S1).

**Table 2:** Impact of pulses and organic amendments on soil constituents (mass–volume relationship).

Depth	Cropping system	Water holding capacity (%)	Gravimetric moisture content (%)	Volumetric moisture content (%)	Volume expansion (cm^3^)
0–20 cm	M–W	38.2 ± 0.45^c#^	34.7 ± 0.12^c^	44.4 ± 0.12^b^	13.9 ± 0.10^b^
	M–W–Mb	39.6 ± 0.22^b^	37.4 ± 0.05^ab^	47.1 ± 0.16^a^	15.2 ± 0.44^b^
	M–W–M–C	40.4 ± 0.16^b^	36.3 ± 0.48^b^	44.5 ± 0.47^b^	14.5 ± 0.31^b^
	P–W	42.0 ± 0.15^a^	38.2 ± 0.17^a^	46.4 ± 0.25^a^	17.3 ± 0.28^a^
	*Nutrient management*				
	CT	37.8 ± 0.02^c^	33.3 ± 0.02^c^	42.5 ± 0.13^c^	12.3 ± 0.30^c^
	RDF	39.8 ± 0.27^b^	37.1 ± 0.05^b^	46.3 ± 0.34^b^	15.0 ± 0.20^b^
	INM	42.6 ± 0.16^a^	39.6 ± 0.13^a^	48.0 ± 0.37^a^	18.3 ± 0.41^a^
	Interaction	*	*	*	NS
20–40 cm	*Cropping system*				
	M–W	38.9 ± 0.39^b^	39.6 ± 0.06^c^	54.0 ± 0.02^b^	16.9 ± 0.36^a^
	M–W–Mb	40.0 ± 0.22^ab^	40.0 ± 0.13^c^	52.8 ± 0.02^c^	18.0 ± 0.21^a^
	M–W–M–C	41.1 ± 0.18^a^	41.4 ± 0.22^b^	54.0 ± 0.07^b^	16.8 ± 0.11^a^
	P–W	41.3 ± 0.24^a^	42.2 ± 0.11^a^	56.0 ± 0.31^a^	17.9 ± 0.13^a^
	*Nutrient management*				
	CT	38.6 ± 0.15^c^	39.3 ± 0.39^b^	53.3 ± 0.51^b^	16.5 ± 0.44^a^
	RDF	40.1 ± 0.12^b^	40.3 ± 0.09^b^	53.5 ± 0.10^b^	17.4 ± 0.23^a^
	INM	42.4 ± 0.22^a^	42.8 ± 0.25^a^	55.8 ± 0.18^a^	18.1 ± 0.33^a^
	Interaction	*	*	*	*

^#^Lowercase letters (a–c) after values (mean ± standard error) delineates significant difference at *p* ≤ 0.05 using Tukey's honest significance test; *denotes interaction is significant; NS = non-significant.

### Volume expansion, specific volume and porosity

Pulse-based rotation significantly increased these parameters over M–W (Table 2; Supplementary Table S1). Specifically, P–W and M–W–Mb rotation had 9–24% and 7–9% higher (*p* < 0.05) volume expansion and porosity over the M–W, respectively. INM increased by 5% and 4% porosity than RDF (*p* < 0.05) across depth. The substitution of wheat with chickpea (M–W–M–C) increased by 9% porosity over M–W (*p* < 0.05) (Supplementary Table S1).

### Soil organic carbon and available nutrients in bulk soil

P–W and M–W–Mb rotations increased soil organic carbon by 10–13% (*p* < 0.05) and 12–18% (*p* < 0.05) across depth over M–W rotation after 14 years of cropping (Table 3). P–W, M–W–Mb, and M–W–M–C increased (*p* < 0.05) available nitrogen (6–11%), available phosphorus (42–73%), and available potassium (8–16%) over the M–W at 0–20 cm depth. The INM resulted in 11–18% (*p* < 0.05) higher SOC over the RDF across soil depths (Table 3). The INM increased 15% available phosphorus over RDF at 0–20 cm depth.

**Table 3:** Impact of pulses and organic amendments on soil chemical properties after 14 years.

Depth	Cropping system	pH	Soil organic carbon (g kg^−1^)	Nitrogen (mg kg^−1^ dry soil)	Phosphorus (mg kg^−1^ dry soil)	Potassium (mg kg^−1^ dry soil)
0–20 cm	M–W	8.13 ± 0.01^a#^	3.82 ± 0.03^b^	103.6 ± 0.4^b^	5.27 ± 0.21^c^	42.2 ± 0.9^b^
	M–W–Mb	8.07 ± 0.04^a^	4.19 ± 0.26^a^	111.5 ± 1.4^a^	7.51 ± 0.26^b^	45.4 ± 0.6^ab^
	M–W–M–C	8.08 ± 0.02^a^	4.22 ± 0.06^a^	110.3 ± 1.7^a^	8.01 ± 0.27^b^	49.0 ± 2.0^a^
	P–W	8.09 ± 0.02^a^	4.33 ± 0.03^a^	114.7 ± 0.9^a^	9.13 ± 0.21^a^	47.1 ± 0.5^ab^
	*Nutrient management*					
	CT	8.18 ± 0.02^a^	3.88 ± 0.05^b^	100.7 ± 0.4^b^	6.63 ± 0.21^b^	41.4 ± 0.3^b^
	RDF	8.05 ± 0.02^b^	3.91 ± 0.02^b^	116.3 ± 1.4^a^	7.37 ± 0.25^b^	47.4 ± 1.3^a^
	INM	8.06 ± 0.01^b^	4.64 ± 0.11^a^	113.1 ± 0.9^a^	8.47 ± 0.14^a^	48.9 ± 0.7^a^
	Interaction	*	*	*	*	*
20–40 cm	*Cropping system*					
	M–W	8.10 ± 0.02^a^	2.77 ± 0.05^b^	76.8 ± 0.4^b^	6.97 ± 1.3^a^	38.1 ± 1.3^a^
	M–W–Mb	8.04 ± 0.01^b^	3.02 ± 0.07^ab^	87.1 ± 2.7^a^	7.38 ± 0.2^a^	39.1 ± 1.7^a^
	M–W–M–C	8.13 ± 0.01^a^	3.27 ± 0.09^a^	80.9 ± 0.8^ab^	7.06 ± 0.6^a^	41.8 ± 2.2^a^
	P–W	8.09 ± 0.03^ab^	3.12 ± 0.16^ab^	84.5 ± 1.8^a^	7.93 ± 0.7^a^	41.3 ± 1.4^a^
	*Nutrient management*					
	CT	8.09 ± 0.02^ab^	2.51 ± 0.06^c^	75.2 ± 0.6^b^	6.01 ± 0.7^a^	36.9 ± 0.2^c^
	RDF	8.10 ± 0.03^a^	3.14 ± 0.07^b^	87.1 ± 2.9^a^	8.27 ± 0.2^a^	39.7 ± 1.0^b^
	INM	8.08 ± 0.01^b^	3.50 ± 0.08^a^	84.7 ± 0.9^a^	7.73 ± 1.3^a^	43.6 ± 0.1^a^
	Interaction	*	*	*	NS	*

^#^Lowercase letters (a–c) after values (mean ± standard error) delineates significant difference at *p* ≤ 0.05 using Tukey's honest significance test; *denotes interaction is significant; NS = non-significant.

### Aggregate stability and aggregated N and P content

**Figure 1:**
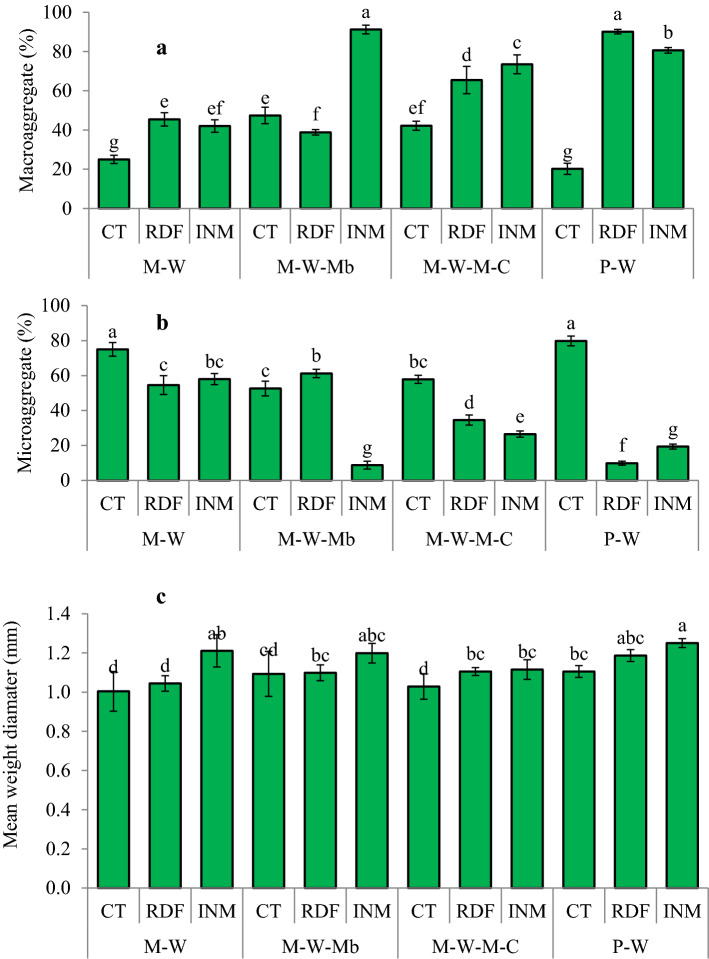
Impact of management practices on aggregate properties after 14 years; lowercase letters (a–g) delineates significant difference at *p* ≤ 0.05 using Tukey's honest significance test.

P–W (INM), P–W (RDF), and M–W–Mb (INM) significantly enhanced the water-stable macroaggregates by 83%, 92%, and 78% over M–W (RDF) (Fig. 1). Subsequently, these rotations decreased 64.4–83.8% water-stable microaggregates than M–W (RDF) after 14 years. The pulse-based system with RDF/INM increased mean weight diameter over M–W (RDF) at 0–20 cm soil depth. Macroaggregated nitrogen was higher in M–W–M–C, P–W, and M–W–Mb by 31%, 40%, and 56% (*p* < 0.05) and microaggregated nitrogen by 13%, 18%, and 36% (*p* < 0.05) over M–W, respectively (Table 4). Similarly, these systems improved macroaggregated phosphorus (7–11%) and microaggregated phosphorus (4–12%) than M–W. INM increased nitrogen content in macro- and micro-aggregates by 10% over the RDF (*p* < 0.05). INM enhanced 20–22% macro- and micro-aggregated phosphorus compared with RDF (*p* < 0.05). Notably, M–W–Mb (INM), P–W (RDF), and P–W (INM) significantly increased nitrogen and phosphorus content in aggregates over M–W (RDF) (Table 4).

**Table 4:** Impact of pulses and organic amendments on aggregated nitrogen and phosphorus content (mg kg^−1^ dry soil).

	Macro-aggregated nitrogen	Micro-aggregated nitrogen	Macro–aggregated phosphorus	Micro-aggregated phosphorus
Cropping system				
M–W	61.0 ± 2.0^c#^	47.4 ± 0.3^c^	6.11 ± 0.13^b^	5.41 ± 0.05^c^
M–W–Mb	85.2 ± 0.9^b^	55.7 ± 1.7^b^	6.66 ± 0.07^a^	5.83 ± 0.02^ab^
M–W–M–C	79.8 ± 0.4^b^	53.7 ± 0.8^b^	6.56 ± 0.03^a^	5.63 ± 0.05^bc^
P–W	95.0 ± 0.7^a^	64.5 ± 0.9^a^	6.80 ± 0.02^a^	6.05 ± 0.10^a^
Nutrient management				
CT	66.9 ± 0.1^c^	45.9 ± 0.3^c^	5.51 ± 0.08^c^	5.05 ± 0.01^b^
RDF	82.6 ± 1.5^b^	57.2 ± 0.6^b^	6.38 ± 0.09^b^	5.46 ± 0.17^b^
INM	91.2 ± 1.7^a^	62.2 ± 0.7^a^	7.71 ± 0.07^a^	6.68 ± 0.04^a^
Cropping system × nutrient management				
M–W (CT)	57.0 ± 1.0^c^	43.8 ± 1.0^d^	4.24 ± 0.22^e^	5.02 ± 0.02^cd^
M–W (RDF)	49.0 ± 0.9^c^	52.7 ± 0.9^c^	6.14 ± 0.16^d^	5.13 ± 0.22^cd^
M–W (INM)	77.0 ± 3.7^b^	45.6 ± 0.8^d^	7.95 ± 0.05^a^	6.07 ± 0.04^b^
M–W–Mb (CT)	70.7 ± 0.7^b^	47.6 ± 2.8^cd^	5.86 ± 0.14^d^	5.02 ± 0.07^cd^
M–W–Mb (RDF)	95.6 ± 2.3^a^	45.6 ± 0.8^d^	6.68 ± 0.07^e^	5.66 ± 0.12^bc^
M–W–Mb (INM)	89.2 ± 4.5^a^	73.8 ± 3.0^a^	7.59 ± 0.06^ab^	6.81 ± 0.11^a^
M–W–M–C (CT)	49.0 ± 0.8^c^	42.0 ± 2.8^d^	5.96 ± 0.16^d^	4.73 ± 0.04^d^
M–W–M–C (RDF)	91.0 ± 4.2^a^	53.9 ± 3.6^c^	6.38 ± 0.29^cd^	5.24 ± 0.33^c^
M–W–M–C (INM)	99.3 ± 5.6^a^	65.2 ± 0.9^b^	7.33 ± 0.23^b^	6.92 ± 0.22^a^
P–W (CT)	91.0 ± 0.5^a^	50.2 ± 0.2^cd^	6.11 ± 0.09^d^	5.42 ± 0.07^c^
P–W (RDF)	94.8 ± 1.1^a^	76.7 ± 1.7^a^	6.32 ± 0.16^cd^	5.80 ± 0.09^bc^
P–W (INM)	99.3 ± 1.3^a^	66.6 ± 0.6^b^	7.97 ± 0.07^a^	6.92 ± 0.22^a^

^#^Lowercase letters (a–d) after values (mean ± standard error) delineates significant difference at *p* ≤ 0.05 using Tukey's honest significance test; *denotes interaction is significant; NS = non-significant.

### Biological properties in the soils

**Figure 2:**
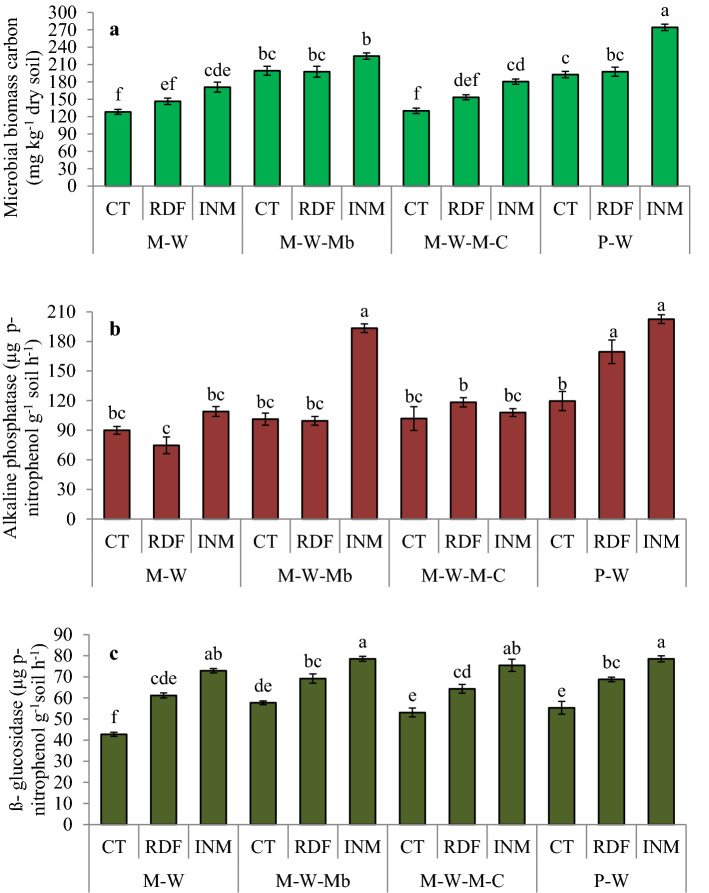
Impact of management practices on soil biological properties; lowercase letters (a–f) delineates significant difference at *p* ≤ 0.05 using Tukey's honest significance test.

P–W (INM) and M–W–Mb (INM) increased (*p* < 0.05) soil microbial biomass carbon by 75–113%, alkaline phosphatase by 114–125%, and β-glucosidase by 83% over M–W (RDF) (Fig. 2). Notably, RDF in each crop rotation had a reduced enzymes activity than that in INM (*p* < 0.05). The CT and RDF under the M–W system had similar alkaline phosphatase content over time.

### Crop productivity and sustainable yield index

**Figure 3:**
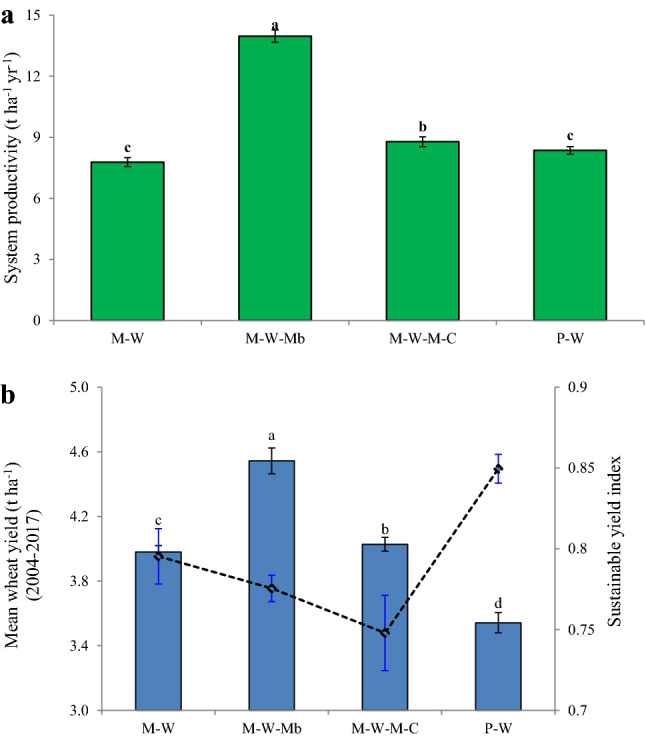
Impact of cropping sequence on yields over time; lowercase letters (a–c) delineates significant difference at *p* ≤ 0.05 using Tukey's honest significance test.

M–W–Mb increased 26% maize yield and 21% wheat yield over M–W (*p* < 0.05) in the 13th crop cycle (2015–2016) (Table 5). Similarly, it increased 23% maize yield and 8% wheat yield over M–W (*p* < 0.05) in the 14th crop cycle (2017–2018). This increasing trend of maize and wheat yields under M–W–Mb has also been observed in the 11th (2013–2014) and 12th (2014–2015) crop cycles. Notably, M–W–Mb increased system yield by 127% (2015–2016) and 80% (2016–2017) over the M–W (*p* < 0.05) (Table 5; Fig. 3a). Even, M–W–M–C resulted in higher yields of maize (19.4%), wheat (12.5%), and system (9–15%) over M–W (*p* < 0.05). The P–W system had 23.7% higher system productivity than the M–W system in 2016–17. The mean yield of 14 years (2004–2005 to 2016–2017) revealed higher wheat yield (base crop) under M–W–Mb and higher sustainable yield index of wheat under P–W (Fig. 3b). INM had higher yields of chickpea (14%) and mungbean (12%) over the RDF (Table 5).

**Table 5:** Grain yields (t ha^−1^) in 2013–2014, 2014–2015, 2015–2016 and 2016–2017 (11th–14th crop cycle) in different systems.

Year	Treatment	Maize	Pigeonpea	Wheat	Chickpea	Mungbean
2013–2014	M–W	3.17 ± 0.09^c#^	–	4.39 ± 0.09^b^		–
	M–W–Mb	3.74 ± 0.08^a^	–	4.62 ± 0.09^a^		1.14 ± 0.03
	M–W–M–C	3.54 ± 0.11^b^	–	–	2.22 ± 0.07	–
	P–W	–	1.47 ± 0.07	3.62 ± 0.12^c^		–
	CT	1.50 ± 0.08^b^	0.80 ± 0.03^c^	1.32 ± 0.06^b^	0.90 ± 0.04^c^	0.90 ± 0.03^b^
	RDF	2.37 ± 0.15^a^	0.90 ± 0.02^b^	2.62 ± 0.06^a^	1.01 ± 0.06^b^	1.21 ± 0.05^a^
	INM	2.28 ± 0.11^a^	1.02 ± 0.05^a^	2.55 ± 0.02^a^	1.16 ± 0.01^a^	1.32 ± 0.02^a^
2014–2015	M–W	3.89 ± 0.07^c^	–	3.96 ± 0.10^b^	–	–
	M–W–Mb	4.55 ± 0.05^a^	–	4.38 ± 0.09^a^	–	1.26 ± 0.02
	M–W–M–C	4.19 ± 0.11^b^	–	4.16 ± 0.09^b^	–	–
	P–W		1.42 ± 0.08	3.64 ± 0.05^c^	–	–
	CT	1.35 ± 0.08^b^	0.78 ± 0.03^c^	2.18 ± 0.06^b^	-	1.13 ± 0.03^b^
	RDF	2.38 ± 0.15^a^	0.93 ± 0.02^b^	3.41 ± 0.06^a^	-	1.28 ± 0.05^a^
	INM	2.17 ± 0.11^a^	1.06 ± 0.05^a^	3.48 ± 0.02^a^	–	1.38 ± 0.02^a^
2015–2016	M–W	1.85 ± 0.07^b^	–	2.00 ± 0.06^b^	–	–
	M–W–Mb	2.34 ± 0.10^a^	–	2.42 ± 0.07^a^	–	1.22 ± 0.03
	M–W–M–C	1.86 ± 0.08^b^	–	–	1.03 ± 0.03	–
	P–W	–	0.90 ± 0.01	1.79 ± 0.0.02^b^	–	–
	CT	1.50 ± 0.08^b^	0.88 ± 0.03^a^	1.32 ± 0.06^c^	0.90 ± 0.04^b^	0.90 ± 0.03^c^
	RDF	2.37 ± 0.15^a^	0.90 ± 0.02^a^	2.63 ± 0.05^a^	1.05 ± 0.06^ab^	1.02 ± 0.05^b^
	INM	2.17 ± 0.11^a^	0.92 ± 0.02^a^	2.26 ± 0.02^b^	1.13 ± 0.01^a^	1.16 ± 0.02^a^
2016–2017	M–W	2.83 ± 0.10^b^	–	2.39 ± 0.08^b^		–
	M–W–Mb	3.48 ± 0.03^a^	–	2.59 ± 0.06^a^		1.03 ± 0.05
	M–W–M–C	3.38 ± 0.06^a^	–	2.69 ± 0.04^a^		–
	P–W	–	0.93 ± 0.05	2.02 ± 0.02^c^		–
	CT	2.12 ± 0.12^b^	0.90 ± 0.06^b^	1.71 ± 0.02^c^		0.94 ± 0.06^c^
	RDF	3.99 ± 0.09^a^	1.06 ± 0.06^a^	2.61 ± 0.06^b^		1.01 ± 0.05^b^
	INM	3.60 ± 0.06^a^	1.06 ± 0.03^a^	2.95 ± 0.02^a^		1.12 ± 0.05^a^

^#^Lowercase letters (a–c) after values (mean ± standard error) delineates significant difference at *p* ≤ 0.05 using Tukey's honest significance test; *denotes interaction is significant.

## Discussion

### Role of pulses on soil properties

Soil compaction in tillage-intensive M–W rotation^45^ could restrict crop/root growth and productivity^6^, which is an evident/pervasive problem in the IGP^33^. Reduction in bulk density is essential for enhancing soil health and crop productivity in the regions. Higher macroaggregate in pulse-based cropping systems reduced bulk density and increased porosity, air ratio, and mean weight diameter in the present study. Tillage operations were similar in all crop rotations in the present study. Hence, variable soil properties were because of the inclusion of pulse crops in the cereal-cereal system (M–W), the deep root system of pulse crops, higher root activities, and leaf fall. A previous study indicated that pulse crops (mungbean, chickpea, and pigeonpea) in rotation increased macropores and macroaggregates because of the decomposition of leaf litter fall, root biomass, and rhizodeposition^46^. The ligno-protein and polysaccharide complexes from fresh leaves and lower carbon:nitrogen ratio of residues of pulse crops increase the soil-aggregate cohesion and aggregate stability (mean weight diameter), thereby reducing the bulk density in the long run^33^. The low molecular weight organic acids and root exudates secreted from pulse rhizosphere could play a crucial role in soil aggregation^21^. In this regard, the pigeonpea crop under the P–W system had higher leaf fall in combination with deep root system and bioturbation (biological tillage) activities that resulted in reduced bulk density and higher physical properties such as soil aggregation, porosity, and air ratio^8^. Added organic matter through leaf fall and root biomass in pulse-based systems could build-up humus that restored soil porosity and aeration in compacted soil^47^. The intensification of the maize–wheat system with mungbean resulted in added crop residue (3 crops year^−1^) and belowground biomass and increased soil aggregation and porosity over maize–wheat^8^.

Higher moisture (gravimetric and volumetric) content and water holding capacity with the inclusion of pulses and INM than chemical fertilization in M–W [M–W (RDF)] could be due to higher SOC that retained soil moisture in these systems. Water-filled pore space was reduced under pulse-based systems than under M–W. The ecological significance of lower water filled pore space is the reduced greenhouse gas emission (specifically carbon dioxide). Microbial respiration, which returns carbon to the atmosphere, can be higher with higher water-filled pore space^48^. The higher water-filled pore space creates anaerobic conditions in the root zone, which generates nitrous oxide emissions. The higher soil aggregation and porosity create aerobic conditions and release nitrous oxide into the atmosphere^47^. In this regard, pulse-based rotations could minimize nitrous oxide emission over the maize–wheat, which had a higher water-filled pore space. Besides, higher air-filled porosity and lower water filled porosity in pulse-based systems can stabilize microbial carbon and minimize CO_2_ emissions^44^.

Pulse-based cropping systems increased aggregated N and P content and available nutrients in the present study. It is because of added carbon and nitrogen through crop residues and rhizospheric alteration by pulse crops. The higher aggregated N and P content and available nutrients are the results of increased nutrient stock in aggregates (N and P) and better solubility of nutrients in M–W–Mb (INM), P–W (INM) and M–W–M–C (INM) over M–W (RDF). The acidification in the root zone during biological nitrogen fixation and mineralization of added organic matter increased the nutrient availability in the mineral fraction of soil with alkaline soil pH (pH 8.1 in the present studied soil)^9^. The average nutrient concentrations in crop residues were 1.03% N, 0.21% P, 1.12% K in rice, 1.48% N, 0.23% P, 0.87% K in wheat, 1.80% N, 0.27% P, 0.99% K in chickpea and 2.14% N, 0.22% P, 0.52% K in mungbean. The higher nutrients inputs through crop residues in pulse-based systems (P–W, M–W–Mb, M–W–M–C) resulted in higher aggregated and available nutrients over time. A lower C/N ratio of pulse crop residues (pigeonpea, chickpea, and mungbean), the additional residue of mungbean crop (under M–W–Mb), higher leaf fall (under P–W system) and acidification in root zone had significant contributions in nutrients availability/solubility^15^. Soil water content and temperature have a crucial role in the sequestration of nutrients in the cropping system^47^. The higher water-holding capacity and soil moisture content in P–W, M–W–Mb, and M–W–M–C systems resulted in higher aggregated nutrient content^5^.

P–W (INM) and M–W–Mb (INM) increased SOC and soil microbial biomass carbon over the M–W (RDF) because of higher carbon input through organic amendments (farmyard manure and biomass of above-ground crop residues returning into the soil)^8^. Long-term inclusion of pulses in the cropping system increased SOC and soil microbial biomass carbon over the M–W system due to the enhanced crop growth, higher crop residue addition, rejuvenation of rhizosphere with diversified microbes, and carbon-rich substrates addition into the soil^44^. The increased substrate availability in a pulse-based system increased microbe abundance (bacteria, fungi, and actinomycetes)^15^. Pulse crops enhanced the SOC and SMBC, which acted as substrates for microbial proliferation, thereby enriching the soil enzymes activity such as alkaline phosphatase and β-glucosidase. The increased activities of soil enzymes are a good indicator of soil health and components for sustainable ecology. Hence, crop diversification with pigeonpea and mungbean as P–W (INM) and M–W–Mb (INM) can be a good management practice for higher soil physical health, SOC sequestration, and enzyme activity in long run.

### Impact of fertilization practices

**Figure 4:**
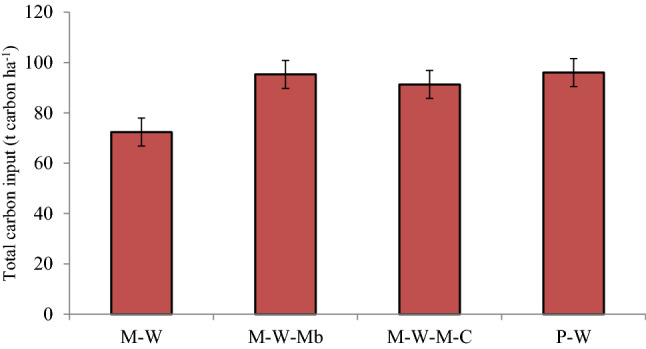
C input through above-ground dry biomass in 14 years.

Added organic amendments in INM under all pulse-based cropping systems reduced bulk density to a greater extent than chemical fertilization in the M–W system. The reduced bulk density in INM practice was because of differential densities of added organic amendments (crop residues and farmyard manure). The dilution effect from mixing of added organic matter reduced bulk density in the mineral fraction of soil having alkaline soil pH. Besides, added organic amendments and their decomposition products could increase microbial activity that favors more aggregation and thus reduce bulk density. Soinne et al. highlighted the higher improvement of bulk density under farm-yard manure/biochar over chemical fertilization^49^. In the present study, macroaggregate was increased under INM in pulse-based systems because of better soil flocculation, and chelating agents that bind the soil^50^. The regulating factors of soil water holding capacities as porosity and specific surface area were regulated by long-term fertilization^21^. Increased SOC under INM also enhanced macroaggregates and mean weight diameter^8^. Total carbon input through organic amendments (farmyard manure and biomass of above-ground crop residues returning to soil) was 88.8 t ha^−1^ in INM (average of crop rotations) in the present study (Fig. 4). On average farmyard manure contained 0.56% nitrogen, 0.18% phosphorus and 0.52% potassium in the present study. It resulted in the addition of 392 kg N, 126 kg P, and 364 kg K ha^−1^ through farmyard manure in 14 years in INM treatment. The total carbon input (FYM + crop residue) in thirteen years in different crop rotations followed the order of P–W (96.0 Mg carbon ha^−1^) > M–W–Mb (95.3 Mg carbon ha^−1^) > M–W–M–C (91.3 Mg carbon ha^−1^) > M–W (72.4 Mg carbon ha^−1^) (Fig. 4). The variable biomass production of crops under study created the difference in added organic amendments (FYM and crop residue). The added carbon into the soil increased aggregate stability and SOC, and resulted in higher gravimetric and volumetric moisture content. Possibly, a reduced soil enzyme activity (β-glucosidase and soil phosphatase) and microbial biomass carbon under RDF minimized aggregate stability^15^. Thus, INM consisting of farmyard manure and crop residues could increase soil aggregation, physical properties, and available nutrients over chemical fertilization in the long run.

### Benefit of carbon and water in soils/aggregation processes

The increased aggregate stability and reduced bulk density in pulse-based systems may be due to the higher SOC concentration^51^. The better pore size distribution and aggregation increased water holding capacity at higher tension under INM treatment than RDF. The higher specific surface area of organic amendments in INM increased water holding capacity and soil moisture content than RDF^52^. P–W and M–W–Mb had an increasing trend of carbon over the year. The increased SOC in P–W and M–W–Mb systems resulted in higher soil biogeochemical properties. Pulses contributed to carbon sequestration with more root biomass than cereals^47^. Thus, higher water holding capacity, gravimetric/volumetric moisture content, and volume expansion in pulses systems contributed to the high carbon input through root biomass that ultimately increased the carbon sequestration potential. The reduced SOC, restricted root zone at the soil surface of cereal crops, low microbial activity, and reduced biomass input in soil over time resulted in the disintegration of soil aggregates in tillage intensive M–W system under RDF. Microbial biomass carbon acted as a chelating agent in the soil aggregation processes and soil moisture retention^47^. Thus, a higher carbon could stabilize soil aggregates and soil water holding capacity in pulse-based systems under INM.

### Implications of pulse-based system for yield sustainability in the region

The present study deciphered that M–W system intensification with mungbean and diversification with pigeonpea/chickpea could restore soil physical health and enhance the yield of crops. The M–W–Mb increased base crop yield (wheat) over the year. It is vital for yield sustainability and food security in the IGP^8, 53^. Wheat is a predominating crop in these regions, where the yield decline of crops is a concern. Further, M–W–Mb and P–W increased system productivity (wheat equivalent yield) over M–W. The increased system productivity could be due additional yield of mungbean in M–W–Mb and the higher price of pigeonpea in the P–W system. Although inorganic fertilizer had a similar grain yield to cereal component crops (maize, wheat) with INM, RDF resulted in a limited effect on soil physical properties. The present study deciphered that intensification of the maize–wheat system with mungbean and diversification with pigeonpea and chickpea could restore the soil's physical, chemical, and biological health in the long run. The higher SOC, soil microbial biomass carbon, β-glucosidase, water holding capacity, available nutrients, and aggregated P could contribute towards yield maximization over time. The advantage of pulse crops in cropping system along with INM [P–W (INM) and M–W–Mb (INM)] can be related to more mineralizable N in pulse crop residues, more residual water in the subsoil, and general rotational advantages of having different preceding crop type^27^. Another benefit of pulses is that they fix atmospheric N_2_ by root nodule symbiosis, and slow release of N from pulse residues and roots favors the growth of succeeding crops and yields^44^.

It is evident that volume expansion, gravimetric moisture content, water-stable macroaggregate, macroaggregated P, air ratio, and soil porosity (%) significantly correlated with wheat yield after 14 years of cropping (Table 6). It indicated that not all parameters could equally contribute to crop yield maximization. Aggregate stability, porosity, and soil moisture content had the higher impact on yield. Hence, management practices that increase soil porosity and aggregate stability could be adopted in tillage-intensive systems. M–W system might increase micro-porosity and soil compaction, restricted root growth, and lower yield. Reversibly, the pulse-based system with INM could rejuvenate aggregate formation, porosity, soil moisture availability, and aggregated nutrient concentration which are vital for crop yield maximization.

**Table 6:** Bivariate regression model on wheat grain yield (t ha^−1^) and soil constituents.

		Parameter (X)	df = n–2	a	slope	t stat	r	r^2^	p value (two-tailed)
0–0.2 m	Grain yield (Y)	Water holding capacity (%)	22	− 0.18	0.07	1.36	0.28	0.08	0.19
		Volume expansion (cm^3^)	22	1.11	0.09	2.08	0.40	0.16	**0.04**
		Gravimetric moisture content (%)	22	− 1.35	0.10	2.91	0.53	0.28	**0.01**
		Air filled porosity	22	1.66	8.54	1.35	0.28	0.08	0.19
		Air ratio	22	1.61	4.45	1.59	0.32	0.10	0.13
		Macroaggregated P	22	0.50	0.13	2.66	0.49	0.24	**0.01**
		Microaggregated P	22	0.85	0.12	1.72	0.34	0.12	0.10
		Water filled pore space (%)	22	1.03	0.02	1.03	0.22	0.05	0.31
		Macroaggregated N	22	1.54	0.01	1.68	0.34	0.11	0.11
		Water stable macroaggregate (%)	22	1.94	0.01	2.03	0.40	0.16	**0.03**
0.2–0.4 m	Grain yield (Y)	Specific volume (m^3^ Mg^−1^)	22	− 8.26	14.2	2.65	0.49	0.24	**0.01**
		Void ratio	22	− 2.86	4.58	2.46	0.46	0.22	**0.02**
		Water holding capacity (%)	22	− 0.17	0.06	1.25	0.26	0.07	0.23
		Porosity (%)	22	− 11.3	0.26	2.89	0.52	0.28	**0.01**
		Air ratio	22	1.79	5.92	1.16	0.24	0.06	0.26
		Liquid ratio	22	− 0.27	2.58	0.61	0.13	0.02	0.55
		Volumetric moisture content (%)	22	4.42	-0.04	− 0.75	− 0.16	0.02	0.46

Significant values are in [bold].

Significant (*p* < 0.05, two-tailed).

## Conclusions

The present study highlighted that the mechanical disintegration of soil under the conventional tilled maize–wheat system of IGP could be ameliorated by pulse inclusion and INM practice in a cropping system. P–W (INM) and M–W–Mb (INM) enhanced soil physical properties: aggregate stability, gravimetric and volumetric moisture content, porosity, air ratio, and chemical properties: soil organic carbon and available nutrients, and soil enzymes activity over time than M–W (RDF). P–W (INM) and M–W–Mb (INM) reduced bulk density and water-filled pore space over M–W after 14 years and could increase soil organic carbon sequestration. Also, these systems increased aggregated N/P content and available nutrients resulting in enhanced soil fertility. The higher soil physical, chemical, and biological properties under pulse-based systems with INM could resulting in higher crop and system productivity over the M–W (RDF). Pre-dominating chemical fertilization proved detrimental to physical and aggregate properties of soil. Notably, P–W (INM) and M–W–Mb (INM) provide carbon substrate into the soil, which enhanced soil aggregate stability and SOC over time. Thus, the present study highlights that sustainable cropping intensification must consist of pulse crops in the cereal dominating agroecologies to minimize soil degradation.

## Supplementary Information

Supplementary Information.

## Data Availability

The datasets used and/or analyzed during the current study available from the corresponding author on reasonable request.
