# Supplementary material for: Long-term impact of pulses and organic amendments inclusion in cropping system on soil physical and chemical properties
Source: Sci Rep. 2023 Apr 20;13:6508. doi: 10.1038/s41598-023-33255-3 (PMC10119138; doi:10.1038/s41598-023-33255-3)
Supplement: Supplementary file 1 — Supplementary Information. [file 41598_2023_33255_MOESM1_ESM.docx]

**Long-term impact of pulses and organic amendments inclusion in cropping system on soil physical and chemical properties**

**Table S1**

Long–term effect of pulse based cropping systems, nutrient management and their interaction on soil physical indices at 0–20 and 20–40 cm soil depths.

| Depth | *Cropping system* | Air filled porosity | Liquid ratio | Specific volume  (cm^3^ g^-1^) | Porosity  (%) |
| --- | --- | --- | --- | --- | --- |
| 0–20 cm | M–W | 0.081±0.003^c#^ | 0.97±0.01^a^ | 0.77±0.003^c^ | 48.8±0.13^c^ |
|  | M–W–Mb | 0.087±0.004^bc^ | 0.90±0.02^bc^ | 0.79±0.002^b^ | 52.2±0.49^b^ |
|  | M–W–M–C | 0.094±0.001^ab^ | 0.87±0.01^c^ | 0.81±0.002^a^ | 53.1±0.46^ab^ |
|  | P–W | 0.101±0.003^a^ | 0.93±0.01^b^ | 0.82±0.001^a^ | 53.4±0.27^a^ |
|  | *Nutrient management* | | | | |
|  | CT | 0.073±0.008^b^ | 1.00±0.03^a^ | 0.78±0.001^c^ | 50.5±0.06^b^ |
|  | RDF | 0.088±0.001^b^ | 0.90±0.02^b^ | 0.80±0.005^b^ | 51.8±0.28^b^ |
|  | INM | 0.110±0.003^a^ | 0.85±0.01^c^ | 0.82±0.003^a^ | 53.3±0.19^a^ |
|  | Interaction | NS | NS | * | * |
| 20–40 cm | *Cropping system* | | | | |
|  | M–W | 0.041±0.001^c^ | 1.05±0.02^a^ | 0.73±0.002^a^ | 52.6±0.41^a^ |
|  | M–W–Mb | 0.057±0.003^ab^ | 1.02±0.03^a^ | 0.76±0.003^a^ | 53.8±0.24^a^ |
|  | M–W–M–C | 0.051±0.005^b^ | 1.06±0.04^a^ | 0.77±0.005^a^ | 54.4±0.21^a^ |
|  | P–W | 0.063±0.002^a^ | 1.04±0.01^a^ | 0.75±0.002^a^ | 53.4±0.26^a^ |
|  | *Nutrient management* | | | | |
|  | CT | 0.048±0.003^b^ | 1.04±0.01^b^ | 0.74±0.001^b^ | 52.6±0.08^c^ |
|  | RDF | 0.045±0.004^b^ | 1.06±0.03^a^ | 0.75±0.002^b^ | 53.5±0.04^b^ |
|  | INM | 0.066±0.002^a^ | 1.02±0.02^c^ | 0.77±0.002^a^ | 54.5±0.06^a^ |
|  | Interaction | NS | NS | NS | * |

^#^within a column, values (mean ± standard error) followed by different lowercase letters (a–c) are significantly different between treatment at *p*≤0.05 using Tukey's honest significance test; *denotes interaction is significant; NS = non-significant.

**Table S2**

Effect of pulse based cropping systems, nutrient management and their interaction on soil chemical properties at 0–20 cm soil depth in 2011 (at 9^th^ year crop cycle).

| *Cropping system* | pH | Soil organic carbon  (g kg^-1^) | Nitrogen (mg kg^-1^  dry soil) |
| --- | --- | --- | --- |
| M–W | 8.12±0.03^a#^ | 3.92±0.07^c^ | 99.0±0.2^b^ |
| M–W–Mb | 8.08±0.04^b^ | 4.13±0.13^a^ | 103.7±0.9^a^ |
| M–W–M–C | 8.11±0.05^a^ | 4.07±0.09^b^ | 104.5±0.8^a^ |
| P–W | 8.13±0.01^a^ | 4.14±0.12^a^ | 106.6±0.4^a^ |
| *Nutrient management* |  |  |  |
| CT | 8.16±0.03^a^ | 3.84±0.02^c^ | 103.9±0.4^b^ |
| RDF | 8.07±0.04^b^ | 3.91±0.03^b^ | 104.7±1.4^a^ |
| INM | 8.10±0.01^b^ | 4.46±0.09^a^ | 104.8±0.9^a^ |
| Interaction | * | * | * |

^#^within a column, values (mean ± standard error) followed by different lowercase letters (a–c) are significantly different between treatment at *p*≤0.05 using Tukey's honest significance test; ^*^denotes interaction is significant.

| 0-20 cm | 20-40 cm |
| --- | --- |
|  |  |
|  |  |
|  |  |
| **Figure S1.** Impact of management practices on soil moisture constituents and carbon content; lowercase letters (a–f) delineates significant difference at *p*≤0.05 using Tukey's honest significance test | |
